# eRNA co-expression network uncovers TF dependency and convergent cooperativity

**DOI:** 10.1038/s41598-023-46415-2

**Published:** 2023-11-04

**Authors:** Seungha Alisa Lee, Katla Kristjánsdóttir, Hojoong Kwak

**Affiliations:** Department of Molecular Biology and Genetics, Cornell University, Ithaca, NY 14850 USA

**Keywords:** Regulatory networks, Transcriptional regulatory elements

## Abstract

Enhancer RNAs (eRNAs) are non-coding RNAs produced by transcriptional enhancers that are highly correlated with their activity. Using a capped nascent RNA sequencing (PRO-cap) dataset in human lymphoblastoid cell lines across 67 individuals, we identified inter-individual variation in the expression of over 80 thousand transcribed transcriptional regulatory elements (tTREs), in both enhancers and promoters. Co-expression analysis of eRNAs from tTREs across individuals revealed how enhancers are associated with each other and with promoters. Mid- to long-range co-expression showed a distance-dependent decay that was modified by TF occupancy. In particular, we found a class of “bivalent” TFs, including Cohesin, that both facilitate and isolate the interaction between enhancers and/or promoters, depending on their topology. At short distances, we observed strand-specific correlations between nearby eRNAs in both convergent and divergent orientations. Our results support a cooperative model of convergent eRNAs, consistent with eRNAs facilitating adjacent enhancers rather than interfering with each other. Therefore, our approach to infer functional interactions from co-expression analyses provided novel insights into the principles of enhancer interactions as a function of distance, orientation, and binding landscapes of TFs.

## Introduction

Transcriptional regulatory elements (TREs), such as promoters and enhancers, are indispensable players in the regulation of gene expression by recruiting transcription factors (TFs)^1–4^. Genes are expressed from promoters where regulatory signals from enhancers are integrated to determine the amount of RNA product. Enhancers act as distinct regulatory elements for promoters at varying distances, which can be relatively proximal to promoters, less than 2 kilobase pairs (kb), or more distal to promoters between genes or within introns. They act through specific TF binding while initiating bidirectional enhancer RNAs (eRNAs). Regulatory networks of TREs involving enhancers and promoters are key to most cellular processes, including development, cell type differentiation, and stress response, while their dysregulation could cause disease^5–10^. Numerous studies have shown that the vast majority of disease-associated genetic variations affect TREs^11–14^. Understanding TRE networks requires knowledge of how TREs interact with each other and the mechanisms by which this regulation is achieved.

The bidirectional production of RNA from TREs, including both enhancers and promoters, is one of the signature hallmarks of regulatory activity in vertebrates^1–4^. Consequently, relying on RNA products to identify TREs emerged as an efficient approach. For instance, the FANTOM5 consortium used Cap Analysis of Gene Expression (CAGE) to generate an atlas of enhancer activity across numerous cell types and tissues^6^. While CAGE is a simple and powerful method for quantifying transcription initiation at genes, it is less efficient for quantifying transcription activity at enhancers, which produce particularly unstable eRNAs. Sequencing methods that capture nascent RNA, such as NET-CAGE or Global Precision nuclear Run-On sequencing with 5′-capped (m^7^G) RNA enrichment (GRO-cap or PRO-cap), measure transcriptional activity directly and are, therefore, better suited for quantifying TRE activity^2, 15^. Transcriptional activity at these transcribed TREs (tTREs), measured by nascent RNA analysis, is a highly robust measure of their regulatory activities.

Once the activity of TREs has been identified and quantified, the focus shifts to determining which specific TREs are responsible for controlling gene expression and the mechanisms by which they do so. Systematic analysis of expression variation can help reveal these targets and mechanisms. Co-expression networks use the variation in expression between different samples to elucidate regulatory circuits^16^. By coupling co-expression analysis with TF binding profiles, the mechanisms of regulatory circuits can be characterized. While physical interactions between TREs, as revealed by chromatin conformation assays^17–20^, remain the gold standard for mapping chromatin interaction networks, functional interactions inferred by co-expression of TRE transcription can complement physical interaction maps to fill in gaps in the regulatory network.

This study exploits the variation in transcription initiation at tTREs, the majority of which are eRNAs^21^. We investigate the interactions between tTREs measured by PRO-cap in lymphoblastoid cell lines (LCLs) from 67 individuals. We use co-variation between tTREs as an indicator of functional interactions between sites, and explore how these interactions are globally influenced by different transcription factors, most of which we validate using 3D chromatin conformation data. We identified thousands of putative interactions and found global signatures of either facilitation or inhibition of interactions for multiple TFs. We also explored interactions between different strands of neighboring tTREs and found evidence suggesting cooperativity at sites with converging transcription, uncovering new rules of eRNA interactions and their potential roles.

## Results

### Co-expression of tTREs as an indicator of correlated association

**Figure 1:**
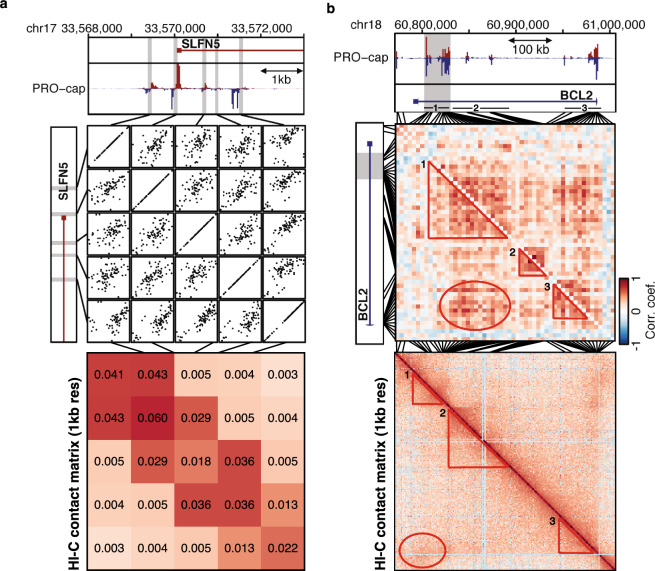
Covariation of tTRE transcription as an indicator of correlated interactions. (**a**) High-resolution example of the co-expression analysis strategy at the SLFN5 locus. The PRO-cap signal at each variably expressed tTRE (proximal enhancer or promoter; grey box) is correlated with the PRO-cap signal of each other variably expressed tTRE in the vicinity, across LCL samples. The lower panel shows the corresponding heatmap of the Hi-C contact matrix with a resolution of 1 kilobase. (**b**) Coexpression over a larger region at the BCL2 locus reveals putative correlated interactions both within and between tTRE clusters (grey box: BCL2 super-enhancer). Heatmap shows correlation coefficients for correlations between tTRE pairs across LCL samples. The lower panel shows the corresponding heatmap of the Hi-C contact matrix with a resolution of 1 kb. The regions of high interaction are highlighted with a circle and three triangles, each representing a different interaction.

We use our previously published PRO-cap datasets in LCLs from 67 Yoruban individuals from Nigeria—International HAP-MAP and 1000 Genome Project cell lines—to study the co-relationship between tTREs using a tTRE co-expression analysis^21^. This dataset contains 87,826 tTREs, of which 12,878 (15%) corresponded to promoters and 74,948 (85%) were defined as putative enhancers. These were identified based on bidirectional transcription of a pair of nascent RNAs within 300 bps of each other. Of these, 29,694 (40%) are variably expressed between individuals using the q-value criteria^21, 22^. As a measure of co-expression, we used Pearson’s correlation coefficients of linear regressions between the PRO-cap signals for pairs of variably transcribed tTREs across individuals. We show two examples in the short range near the *SLFN5* gene promoter and in long range at the *BCL2* super-enhancer locus (Fig. 1a,b). Identifying tTREs at high resolution allows us to hone in on individual tTRE elements and their correlations (Fig. 1a, upper linear regression panel), in contrast to the 1 kilobase (kb) resolution Hi-C data in the representative LCL GM12878 cell line^23^ around this region (Fig. 1a, lower heatmap panel).

The example of the *BCL2* locus illustrates how this analysis allows us to visualize which tTREs are correlated both within clusters of tTREs and between such clusters. For example, the gray-shaded *BCL2* super-enhancer region (triangle 1) and the *BCL2* promoter region (triangle 3) show a positive correlation (red ellipse) (Fig. 1b, upper heatmap panel). However, the other two clusters of intronic enhancers (position 60,850,000–60,900,000, triangle 2) do not show a strong positive correlation with either the *BCL2* super-enhancer or *BCL2* promoter regions. The Hi-C contact matrix reproduces this pattern (Fig. 1b, lower heatmap panel), showing distinct chromatin domains containing the *BCL2* super-enhancer (red triangle 1), two intronic enhancer clusters (red triangle 2), and the *BCL2* promoter region (red triangle 3). We observed an increased contact frequency between the domains 1 and 3 (red ellipse) compared to between 1 and 2 or 2 and 3, confirming our correlative co-expression finding.

**Figure 2:**
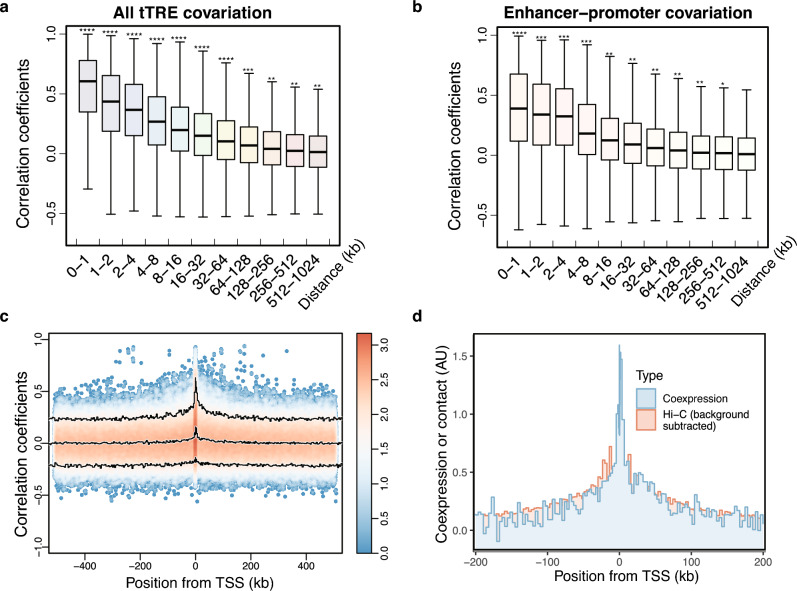
Distance-dependent tTRE co-expression, compared to Hi-C contacts and mRNA PRO-cap co-expression patterns. (**a**) Co-expression of tTREs decreases with increasing distance. tTRE pairs are binned according to the distance between them and the distribution of correlation coefficients in each bin is plotted as a boxplot. ****P < 1 × 10^−4^, and ***P < 1 × 10^−3^, and **P < 1 × 10^−2^ by Wilcoxon rank sum test. (**b**) Covariation between PRO-cap read counts between promoter tTREs and enhancer tTRE (eRNA) expression levels. The distribution of correlation coefficients in each bin is shown as a boxplot. ****P < 1 × 10^−4^, and ***P < 1 × 10^−3^, and **P < 1 × 10^−2^ by Wilcoxon rank sum test. (**c**) Distribution of correlation coefficients between mRNA TSS and tTREs as a function of their relative positions. Pairs were binned based on both the distance between tTRE and mRNA TSS and the orientation of the pair (TRE upstream: negative numbers, tTRE downstream: positive numbers). Density scatter plots show the distribution of the correlation of mRNA expression (RNA-seq^7^) and tTRE expression (PRO-cap), and the 3 lines represent the quantile trajectories of the 5th percentile, median, and 95th percentile of the correlation coefficients. (**d**) Quantile (median) traces of Hi-C contacts compared to mRNA-PRO-cap correlation of co-expression. Traces of background subtracted Hi-C contacts between TSS-tTRE (red) superimposed on the distance-dependent decay of PRO-cap co-expression (blue). The y-axis is in arbitrary units (AU), where 1 unit corresponds to either PRO-cap correlation coefficient of 0.1 or a Hi-C contact frequency difference of 4.5 × 10^−3^.

We investigated whether the co-expression reveals the principles of relationships between tTREs as a function of distance, whether mediated by physical interactions between sites or other mechanisms such as spreading of chromatin modifications or polymerase read-through. When we bin the correlation coefficients based on the distance between tTREs, the covariation of the PRO-cap levels decreases with increasing distance (Fig. 2a). The distribution of the coefficients reaches background levels (comparable to interchromosomal interactions) at 1 Megabases (Mb) (Supplementary Fig. 1a), and the fraction of significantly correlated (FDR < 0.05) tTREs shows a distance-dependent decrease (Supplementary Fig. 1b). A similar trend was observed when we restricted the correlation to promoter-enhancer pairs (Fig. 2b, Supplementary Fig. 1c). Most of the coefficients that exceed the expected distribution for tTRE pairs (either interchromosomal or more than 1 Mb apart) are found in tTRE pairs that are within a maximum distance of 200 kb, and primarily within 100 kb (Supplementary Fig. 1d,e). This distance effect is unlikely to be biased by linkage disequilibrium (LD) of genotypes at tTRE pairs, since the YRI population is known to have LDs smaller than 5 kb^24^ and a similar degree of distance decay is observed using only tTRE pairs with independent genotypes (genotype correlation < 0.05, Supplementary Fig. 1f). The covariation between variably transcribed tTREs and mRNA levels varied depending on the distance between the tTRE and gene promoters or mRNA TSSs (Fig. 2c, Supplementary Fig. 1g).

We also compared this distance-dependent correlation decay with the pattern of Hi-C chromatin contacts in the GM12878 cell line^23^. We reconstructed the contact frequency distributions between the same tTRE pairs where we examined the co-expression correlations, which showed similar distance-dependent decay patterns (Supplementary Fig. 2a–c). In particular, Hi-C contacts between mRNA TSSs and tTREs were higher than the contact frequencies between non-TRE background regions (Supplementary Fig. 2d). While the distance decay of Hi-C contact frequencies continues to decrease beyond 200 kb (Supplementary Fig. 2c), the contact frequency between TSS and tTRE tapered off beyond 200 kb showing a similar decay trend as the mRNA-PRO-cap correlation after subtracting the background Hi-C contact frequencies (Fig. 2d). These comparisons show that the distance-dependent PRO-cap correlation fits well with the Hi-C 3D chromatin contact data.

### TF binding sites at or between tTREs are associated with differences in tTRE co-expression

Binding of transcription factors can alter the interactions between TREs. Insulator proteins such as CTCF can disrupt the communication between two regions^25^ and transcriptional coactivators such as P300 can bridge TREs to their targets^26^. We tested these using published Chromatin Immunoprecipitation (ChIP-seq) peaks (Supplementary Table 2) from ENCODE Factorbook repository^27^ in the representative LCL (GM12878) to determine the effect of TF binding on distance-dependent tTRE correlation trends.

**Figure 3:**
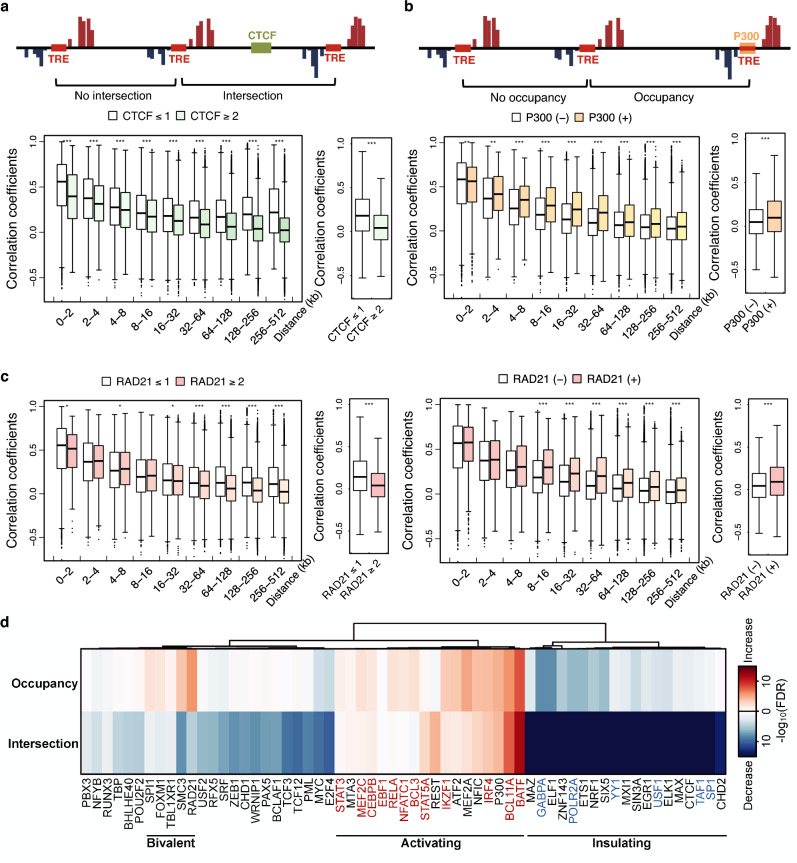
Transcription factor binding sites are associated with changes in tTRE co-expression. (**a**) Intersection by an insulator is associated with decreased tTRE co-expression. Top: Diagram of CTCF crossing the region between two tTREs. Bottom: Distribution of correlation coefficients for tTRE pairs intersected by 2 or more CTCF sites and 1 or less, binned by distance (left) or total (right). ***P < 1 × 10^−3^ by Wilcoxon rank sum test. (**b**) Coactivator occupancy is associated with increased tTRE co-expression. Top: Diagram of P300 occupancy plot of a tTRE. Bottom: Correlation coefficients for tTRE pairs occupied or not occupied by P300, binned by distance (left) or total (right). ***P < 1 × 10^−3^ and **P < 1 × 10^−2^ by Wilcoxon rank sum test. (**c**) Intersection and occupancy by RAD21 have a “bivalent” association with tTRE co-expression. Left: Distribution of correlation coefficients for tTRE pairs intersected by 2 or more RAD21 sites and 1 or less, binned by distance (left) or total (right). Right: Coactivator occupancy increases tTRE co-expression. Correlation coefficients for tTRE pairs occupied or not occupied by RAD21, binned by distances (left) or total (right). ***P < 1 × 10^−3^ and *P < 1 × 10^−1^ by Wilcoxon rank sum test. (**d**) The effect of TF intersection and occupancy on tTRE interactions for 61 TFs. The heat map shows the − log_10_(FDR) for ∆AUC between with and without TF intersection or occupancy (Supplementary Fig. 2b,c). TFs are ordered by minimum variance hierarchical clustering. The number of tTRE pairs in each distance bins in panels (**a**–**c**) are indicated in Supplementary Table 3.

To examine the insulating effect of CTCF on PRO-cap correlations, we separated tTRE pairs into “no intersection” and “intersection” categories based on the number of CTCF ChIP-seq peaks^28^ between them. We plotted the distribution of correlation coefficients as a function of distance (Fig. 3a). Because of the high prevalence of CTCF binding sites, we compared the sets of tTRE pairs that are intersected by 2 or more CTCF binding sites (color-filled box plots), or 1 or less (white box plots). Comparison of tTRE pairs between any CTCF intersection and no intersection yielded similar results, but few tTRE pairs in the no-intersection group in distant bins (Supplementary Fig. 3a). The number of CTCF sites between tTREs is associated with reduced co-expression within each distance bin and overall (Fig. 3a, Supplementary Fig. 3b–d). We also investigated the relationship between coactivator P300 and co-expression by comparing tTRE pairs that are occupied by P300^27^ to those without P300 binding. As expected, P300-bound tTRE pairs have higher levels of co-expression than the unoccupied pairs (Fig. 3b, Supplementary Fig. 3e–g).

While CTCF and P300 showed the same relationship with tTRE correlations, irrespective of their binding mode (intersection or occupancy), RAD21, a subunit of the cohesin complex, shows an “insulating” intersection pattern similar to CTCF (Fig. 3c, left panel), and an “activating” occupancy pattern similar to P300 (Fig. 3c, right panel). These examples show that our co-expression analysis is able to detect the expected effects of TF binding, both when the TF is intersecting and when it is occupying the tTREs.

To further explore these different categories of TF-co-expression relationship, we expanded the analysis to all 60 TFs for which ChIP-seq data were available from the ENCODE repository^27^. To generate a metric of how TF binding is associated with the distance-dependent decay of co-expression, we used the upper 5th percentile trace of the distribution of all correlation coefficients binned by distance (1000 tTRE pairs in each bin) (Supplementary Fig. 1d, top trace). We used the Area Under the Curve (AUC) as a metric to estimate the degree of positive correlation between tTREs within the set of tTRE pairs. We calculated the difference of the AUC between “occupancy”/“no occupancy” and “intersection”/“no intersection”tTRE pairs (∆AUC) (Supplementary Fig. 3b–k). The results from using the top 5th percentile trace correlate well with using either the median trace (Supplementary Figs. 3c–l, 4a,b) or by comparing the proportions of significantly correlated tTREs (FDR < 0.05) in each distance bin (Supplementary Fig. 3^3^–m). We compared the ∆AUC with the permuted background ∆AUC distributions to assess the significance of the difference (Supplementary Fig. 4c,d, see “Methods”).

The TF analysis revealed an extended repertoire of TFs in the three broad categories: insulating, activating, and “bivalent” (Fig. 3d). Insulating TFs, as seen in CTCF, are correlated with reduced tTRE co-expression both when intersecting and occupying tTREs. The opposite is true for activating TFs, including P300. The activating category contains many TFs that are immune or B cell specific (Fig. 3d, red), whereas the insulating category contains general transcription factors associated with strong promoters (Fig. 3d, blue). “Bivalent” TFs, such as the cohesin subunits RAD21 and SMC3, are associated with enhanced covariation when they occupy tTREs, but with repressed covariation when they intersect tTREs. Another bivalent factor, FOXM1, which controls cell cycle progression, is also known to function as both a repressor and an activator depending on the chromatin context^29^.

### TF dependency of tTRE Hi-C contact is consistent with PRO-cap co-expression

**Figure 4:**
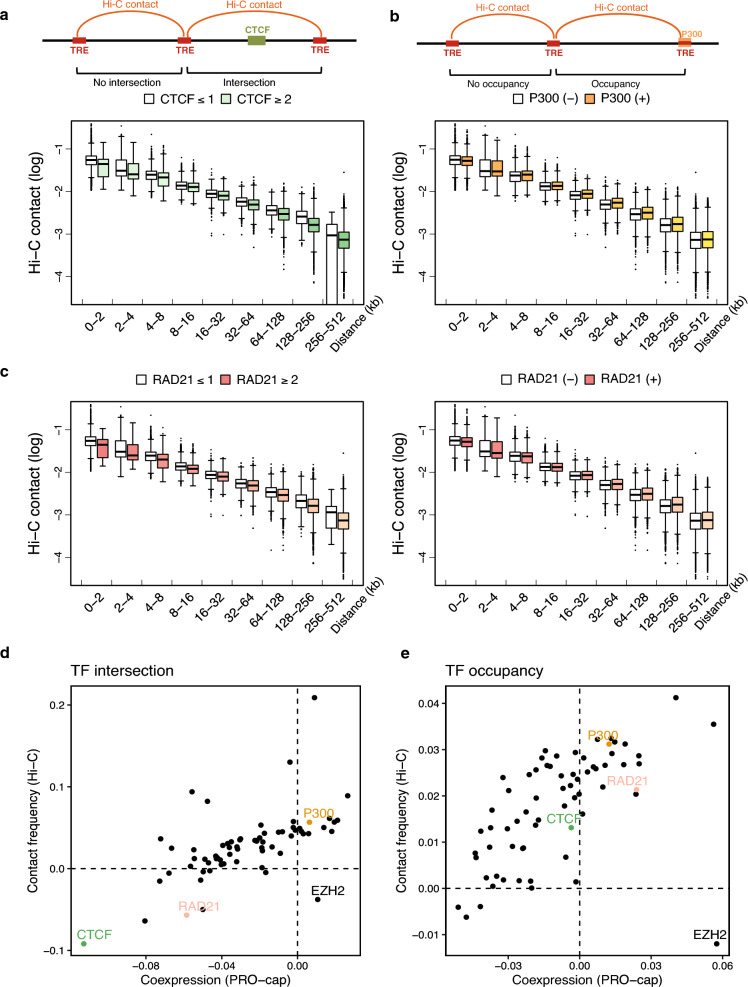
Transcription factor binding sites correlate with variations in the co-expression of tTRE Hi-C contacts. (**a**) Intersection by an insulator is associated with decreased Hi-C contact frequency. Top: Diagram of the intersecting CTCF binding site. Bottom: Distributions of Hi-C contact frequencies for tTRE pairs intersected by 2 or more CTCF sites and 1 or less, binned by distance. (**b**) Coactivator occupancy is associated with increased Hi-C contact frequency. Top: Diagram of P300 occupancy of a tTRE. Bottom: Hi-C contact frequencies for tTRE pairs occupied and unoccupied by P300, binned by distance. (**c**) “Bivalent” association of RAD21 with Hi-C contact frequency depending on tTRE intersection or occupancy. Left: Hi-C contact frequency distributions for tTRE pairs intersected by two or more RAD21 sites and one or less, binned by distances. Hi-C contact frequencies for tTRE pairs occupied or not occupied by RAD21, binned by distances. (**d**) Scatterplot of TF-dependent ∆AUCs comparing PRO-cap coexpression and Hi-C contacts. Each dot represents a TF, and selected TFs are highlighted. Left: ∆AUC scatterplot of TF overlap. Right: ∆AUC scatterplot of TF occupancy. The number of tTRE pairs in each distance bins in panels (**a**–**c**) are indicated in Supplementary Table 3.

Since one of the most important mechanistic links to the correlation of PRO-cap co-expression at tTREs is chromatin contacts, we compared the PRO-cap correlation results with the Hi-C data in the context of TF binding (Fig. 4). We observed a consistent pattern of differences in chromatin contacts based on the intersection and occupancy of transcription factors (TFs). This pattern is in accordance with our correlative co-expression analysis of tTRE transcription. For example, we compared the same sets of tTRE pairs that are intersected by 2 or more CTCF binding sites (color-filled box plots), or 1 or less (white box plots). The Hi-C contact frequencies are overall higher in less intersected sets than in more intersected sets within the same distance bins (Fig. 4a). Similarly, we observed the same Hi-C contact frequency patterns that recapitulate PRO-cap co-expression findings in P300 (Fig. 4b) and RAD21 (Fig. 4c).

To expand this analysis to our TF panel, we plotted the Hi-C contact frequency as a distance-dependent decay step function, as we did for the PRO-cap co-expression analysis, and calculated the ∆AUC in the same way (Supplementary Fig. 5). The ∆AUC metrics for TF intersection and occupancy overall showed a positive correlation between Hi-C and PRO-cap (Fig. 4d,e). This indicates the mechanism behind the relationship between co-expression of tTRE and TF binding is generally based on physical interactions of the chromatin. CTCF shows the lowest ∆AUC in both PRO-cap correlation and Hi-C contact when it intersects two tTREs, consistent with its known role as an insulating factor (Fig. 4d). Likewise, the cohesin complex subunit RAD21 is associated with an insulating effect in both Hi-C contact and PRO-cap co-expression when it intersects tTREs (Fig. 4d), but associates with an increase in both Hi-C contact and PRO-cap co-expression when occupying tTREs (Fig. 4e).

Although there was an overall positive correlation between Hi-C contacts and PRO-cap co-expression, we observed that the Hi-C data mainly showed increased contacts with TF binding. This is supported by the predominantly positive ∆AUC values (horizontal dashed lines) in both intersection and occupancy plots, as depicted in (Fig. 4d,e). Moreover, EZH2, a factor involved in H3K27 methylation and “poised” enhancers^30^, deviated from this trend. This was more evident in the occupancy plot (Fig. 4e, the lower right corner), causing an increased co-expression of PRO-cap but decreased Hi-C contact.

### Strand-specific covariation at adjacent tTREs supports a cooperative model for convergent transcription

**Figure 5:**
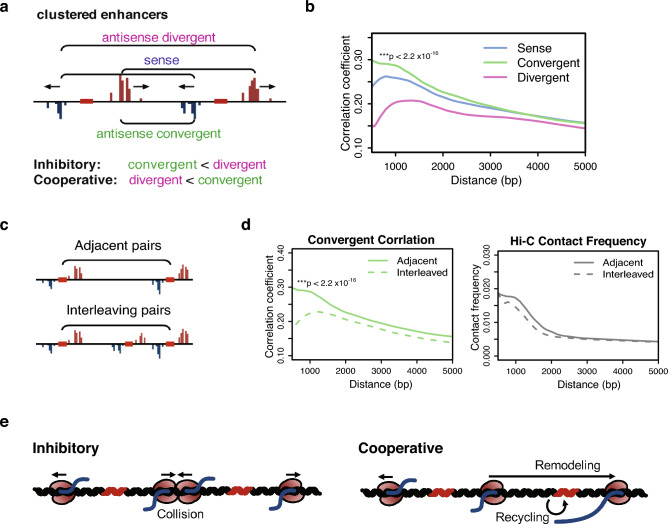
Covariation analysis for convergent and divergent strands of adjacent tTREs. (**a**) Diagram shows three strand comparisons for two adjacent tTREs. Sense comparisons (dark red) are strands transcribed in the same direction, antisense comparisons (blue) are between strands transcribed in opposite directions, with polymerases either converging (green) or diverging (pink). (**b**) LOESS fit of distance-dependent correlation coefficients between sense, convergent, and divergent TSSs (n = 21,486 pairs each); p-value derived by the *t*-test of correlation coefficients within 500–1500 bp bin. (**c**) Diagram of the comparison of co-expression between adjacent and interleaved tTRE pairs. (**d**) LOESS fit for the correlation coefficients (left) and Hi-C contact frequencies (right) of adjacent tTREs directly neighboring each other (solid lines) with interleaved tTREs (dashed lines). Note that adjacent pairs have higher correlation than interleaved pairs, and the p-value is derived by the *t*-test of correlation coefficients within 500–1500 bp bin. (**e**) Diagram of the models for the cooperative convergent transcription of eRNAs versus the inhibitory convergent transcription. Left: Two adjacent enhancers (TREs, red bars) engage two pairs of bidirectionally divergent RNA polymerase II (Pol II, coffee bean shapes filled with red) to transcribe nascent RNAs. Right: Only one of the convergent Pol II is transcribed (from the left enhancer) that passes through.

At close distances, RNA polymerases at one tTRE can potentially affect the adjacent tTRE in either a cooperative or inhibitory manner (Fig. 5a). Recent works have suggested that convergent transcription near promoters and intragenic enhancers attenuates transcription from the gene through polymerase interference^31, 32^. Others have shown that transcriptional read-through leads to increased chromatin accessibility^33–36^ and thus cooperativity. It is also possible that transcription at a site increases the local concentration of RNA polymerase and TFs, either leading to or disrupting the rapid recycling of polymerase from one tTRE to the next^37^.

The strand specificity of PRO-cap allows us to determine which model is more prevalent. If convergent transcription were inhibitory, the converging strands of two adjacent tTREs (upstream plus and downstream minus) would show a lower overall correlation than the diverging strands (upstream minus and downstream plus) and vice versa if convergent transcription were cooperative (Fig. 5a). If the local enrichment of RNA polymerase and TFs mediate the interaction, we would see no difference between the strands. We performed a strand-specific local regression of correlation coefficients for all adjacent tTRE pairs within 10 kb distance (n = 21,486) and compared the distribution of the orientation-specific correlation coefficients (Supplementary Fig. 6a). For closely spaced tTREs (250 bp–1 kb apart), the convergent strand pairs are significantly more correlated than the divergent pairs at the same distance (Fig. 5b). This pattern is also evident when we consider only the enhancer tTREs (Supplementary Fig. 6b), but is no longer evident for tTREs that are further apart and therefore less likely to involve direct read-through (> 5 kb) (Supplementary Fig. 6c). The convergent correlation also decreases when the tTRE pairs are interleaved by another tTRE (Fig. 5c,d, left).

To explore whether chromatin contacts could explain the PRO-cap convergent correlations, we used the Hi-C contact frequency data for the tTRE pairs that are not on the same 1 kb blocks, and determined the effect of interleaved tTREs on the contact frequencies (Fig. 5d, right panel). The Hi-C data do not show significant changes in contact frequencies in the presence of interleaved tTREs, suggesting that the local loop conformation remains unchanged with another tTRE in between. Taken together, these results are more consistent with a model in which convergent transcription is cooperative rather than inhibitory, potentially mediated by Pol II elongation, where direct read-through shows a stronger effect (Fig. 5e).

## Discussion

We made extensive correlational observations of eRNA transcribing tTREs using the variation in transcription initiation across human LCLs. Our results uncovered the principles of co-expression between tTREs dependent on distance, TF binding, and the direction of transcription. Similar to Gene Co-expression Network (GCN) approaches, correlation of tTREs can serve as an important inference of functional interaction. While co-expression does not necessarily indicate a physical interaction, we complemented our analysis with Hi-C analysis and illustrated physical interaction patterns that were broadly consistent with our co-expression conclusions.

The tTREs previously identified by Kristjánsdóttir et al.^21^ using the PRO-cap data served as a critical resource for this study. This deeply sequenced data (~ 1.4 billion unique sequences), using heuristic algorithms to detect the consensus eRNA architecture of enhancers and promoters, allowed the identifications of 76.7 thousand tTREs that were bidirectionally transcribed. This identification of TREs based on capped nascent RNA sequencing provides a direct measure of transcriptional activity that is highly sensitive. A direct measure of transcriptional activity is important, as non-productive transcripts such as eRNAs and uaRNAs are rapidly degraded in the nucleus. Other transcription-based approaches, such as CAGE and nuclear short RNA analysis, are impeded by this instability. As we showed previously^21^, CAGE performs well in identifying promoters but less efficiently detects enhancers than PRO-cap. Additionally, by focusing on bidirectional eRNA transcription start sites (TSS) from PRO-cap data enabled us to filter out spurious transcription from only one strand. Taken together, PRO-cap provides a high-quality set of tTREs that is suitable for large scale correlational analysis.

The co-expression analysis allowed us to explore how TREs interact with one another to form *cis*-regulatory networks. Physical interaction maps have been the standard for identifying direct interactions. However, co-expression network approaches identify correlations that serve as an inference of functional interactions. Though indirect, they provide independent evidence for interactions and complement physical maps. Also, they allow us to infer tTRE interactions mediated by mechanisms that do not necessarily rely on physical interactions but may involve read-through transcription remodeling the chromatin of adjacent sites or shared upstream regulatory mechanisms. We were able to capture mid-range interactions (within 200 kb), which were dependent on distance and TF binding in a manner consistent with current knowledge and validated those results using published Hi-C chromatin conformation data. Interestingly, factors associated with strong promoters were insulating, indicating that strong promoters may dominate functional interactions and repress looping across them. This may be analogous to chromosomal boundaries in *Drosophila* that are formed by paused RNA polymerases^38^.

Comparison between the correlational co-expression of enhancer RNA or tTRE transcription, and physical contact maps may provide further insight. While most of our observations were consistent between PRO-cap co-expression and Hi-C contact frequencies, there were subtle differences. First, Hi-C contacts between tTREs were overall higher (∆AUC > 0 on the y-axis) whenever any TF was bound, even with known insulators or repressors (Fig. 4d,e). This contrasted with the PRO-cap correlation, which showed a decreased correlation (∆AUC < 0 on the x-axis) with these sets of TFs. The increased contact and decreased correlation have a plausible mechanistic explanation for repressors, but is not as consistent with the known function of insulators.

A second outlier from the observed consistency between co-expression and contact is EZH2 (Fig. 4e). EZH2 occupancy is associated with higher tTRE co-expression, but lower Hi-C contact frequency. EZH2 is a H3K27 methyltransferase and is associated with bivalent chromatin and poised enhancers^30^. The discrepancy between Hi-C contact and PRO-cap co-expression can be interpreted as follows: PRO-cap identifies the activated subpopulation of EZH2-bound poised enhancers that are more dynamically associated with gene activation. Meanwhile, Hi-C detects the total population of EZH2-bound enhancers, a majority of which are inactive and remain poised.

The third discrepancy between the two methods is in the short-range local interactions between convergent tTREs that are interleaved by another tTRE (Fig. 5c). While our PRO-cap co-expression shows decreased correlation in interleaved tTREs, Hi-C contact does not decrease significantly (Fig. 5d). If the co-relationship between convergent eRNA transcription is mediated by local 3D structure, we expect to observe a consistent pattern between Hi-C and co-expression data. Our observation of the subtle difference between Hi-C and co-expression in this local context, although not definitive, is evidence against the local loop model of convergent eRNA and towards a Pol II elongation mediated model (Supplementary Fig. 6d).

Thus, while most mid- to long-range interactions occur through DNA looping, the correlations we observed between closely clustered tTREs suggest a transcription-dependent mechanism (Fig. 5e). Previous studies have shown that polymerase collision leads to transcription termination between highly expressed intragenic enhancers and their host genes^31^ (Fig. 5e, inhibitory). However, most tTREs are much less active, making simultaneous transcription and polymerase collisions rare. Instead, our strand-specific co-expression analysis suggests cooperativity rather than inhibition between convergent transcriptions at clustered tTREs (Fig. 5e, cooperative). We speculate that transcription from one tTRE remodels the chromatin architecture in the neighboring region to increase accessibility (Fig. 5e, remodeling). Another possibility is direct recycling of polymerase by termination and reinitiation at a neighboring tTRE, which could be further tested with a more directed transcription termination analysis (Fig. 5e, recycling).

In this study, we demonstrated that using capped nascent RNA sequencing to elucidate the network between transcribed TREs can be a potent tool for exploring gene regulation. Although the pinpointing of individual cis-regulatory networks may face limitations due to the scope of our current study, the principles and insights gleaned from this research will assist in predicting the regulatory targets of TREs, as well as in understanding their biological implications.

## Methods

### Identification of tTREs and selection of variably expressed tTREs

Transcribed Transcriptional Regulatory Elements (tTREs) were identified from 76 partially replicated PRO-cap data in Lymphoblastoid Cell Lines (LCLs) from 67 individuals from the Yoruban population (YRI) as described by Kristjánsdóttir et al.^21^ (Supplementary Table 1). Briefly, we merged all the PRO-cap reads (~ 1.4 billion unique molecular identifiers separated reads) from the dataset that were mapped to the hg19 reference genome. The reads were scanned along the genome to pick out the local maxima within the 300 base window in a strand specific manner. The local maxima peaks were then matched to another local maxima of the opposite strand between 50–250 bases upstream on the antisense direction, so that the paired PRO-cap peaks on both strands form a divergent bidirectional transcription pattern. Single strand peaks without a divergent bidirectional pair were discarded. If there were multiple single strand peaks within the 150 base pair window, we selected the peak with the highest amount of PRO-cap reads. As a result, the closest elements are at least 150 base pairs apart, which is well above our resolution for distinguishing two nascent transcription start sites. We excluded tTREs with ambiguous start sites within 150 bp. The 150 bp cutoff corresponds to one nucleosome distance, which has a structural rationale that enhancers with accessible chromatin have at least one nucleosome removed to create an open chromatin, and eRNA transcription occurs in a bidirectional manner around the boundaries of this region. 89.5% of the tTREs we used were at least 300 bp apart from each other. This process identified 76,730 tTREs that were bidirectionally transcribed.

To identify tTREs that are variably expressed, we used normalized reads-per-million (RPM) normalized PRO-cap read count data containing partial replicates as described previously^21^. Briefly, we used the q-value method described by Storey et al.^22^. We used partially replicated samples as the level of technical variation and used the variation in technical variation as the reference to calculate p-values of pairwise differences between non-replicated different individuals. For each tTRE, we calculated the deviation from the mean of the normalized read counts between replicates and between different samples. We then used a one-sided Wilcoxon’s rank sum test to test the alternative hypothesis that the differences between samples were greater than between the replicates for each tTRE, and calculated p-values. We estimated the number of variably expressed tTREs by analyzing the complete distribution of the p-values as described previously^22^. Under the null hypothesis, p-values should have a uniform distribution with a density of 1, but the observed p-values are only uniformly distributed only for large p-values. The density of the portion of the p-value distribution that is uniform is ~ 0.281, indicating that up to ~ 71.9% of tTREs can be considered variably expressed. Using FDR < 0.2, we identified 29,694 variably expressed tTREs (40% variably expressed).

### Distance-dependent pair-wise co-expression analysis of tTREs

We used the variably expressed tTREs (n = 29,694; promoter—4006; enhancer—25,688 using the CAGE based criteria), and calculated correlation coefficients of the 75 individual normalized read counts (67 individuals + 8 replicates) between two tTREs within 5 Mb distance (2,249,839 pairs). For the distance analysis, we binned the correlation coefficients by the distance between 2 tTREs from all the tTREs. The bins are generated based on fixed distance intervals up to 1024 kb (0–1 kb, 1–2 kb, 2–4 kb, etc.), or a fixed number of tTRE pairs (1000 pairs per bin) with variable distance intervals. All the tTRE pairs were grouped into distance groups, and the box plots were generated to display the median, 25th and 75th percentiles of the correlation coefficients. All of the distance groups in the box plot analyses contained at least 200 tTRE pairs, and the comparison of their means could be expected to follow a Gaussian statistic in Student *t*-tests.

With the variable interval bins of 1000 pairs, we generated plots for the 5th percentile, median and the 95th percentiles of the correlation coefficient distributions within each bin of 1000 pairs along the distance (Supplementary Fig. 1d,e). These percentiles were used to generate a step function of correlation coefficient percentiles as a function of distance. The top 5th percentile is the 50th highest correlation coefficient per bin (of 1000 elements) in this step function analysis, and using the false discovery analysis of correlation p-values, the 5th percentile corresponded to FDR < 0.006. The correlation percentile step function was superimposed on the color density scatterplot of the correlation coefficients for visualization (traced scatterplot) and Area Under the Curve (∆AUC) analysis.

### Estimation of the correlation bias of the spurious correlation and the genotype linkage

To obtain an estimate of spurious correlations, we used 2 million random inter-chromosomal correlations and correlations that are more distant than 1 megabase away as the background distribution of PRO-cap correlations. Both distributions are fitted to the Gaussian distribution with the standard deviation of the correlation distribution and mean of 0, and tested with QQ-plot to indicate their normality using the “qqnorm” function in the R statistical package.

We performed an independent genotype analysis to exclude the possibility that some of the tTRE variation is genetically driven by SNPs and to ensure that the observed correlations were not confounded by the genetic association of the SNPs in the YRI population. To exclude this possibility, we performed the same correlation analysis on tTREs that are not genetically associated (discrete Pearson’s correlation of the genotypes labeled as 0, 1, or 2—reference, heterozygous, alternative alleles). If there were no variable SNPs within the enhancer region, these regions were excluded from the genotype association and considered genotype-independent. Minor allele frequency selection (greater than 0.05) was applied only to the enhancers with variable SNPs to exclude tTREs whose eRNA expression levels were suspected to be genetically associated with SNPs. From this analysis, approximately 5% of tTRE-tTRE pairs were significantly genetically associated by this criterion (FDR < 0.1) and were removed from the analysis. At least 70% of the tTRE-tTRE pairs did not contain any SNPs associated in the population (discrete Pearson correlation less than 0.05, n = 129,660), allowing for more rigorous cut-off of genotype-independence to exclude that genetic linkage in the population confounded the co-expression patterns.

### Co-expression analysis between tTRE nascent transcription and mRNA expression

We used the RNA-seq expression data from Pickrell et al.^7^ which included normalized RNA levels in 161 replicated LCL datasets from the same 67 YRI individuals that we used in PRO-cap. We selected 13,002 genes with the mean expression levels greater than 1 RPKM. 275,660 pairs of tTREs and annotated mRNA TSS within 1 Mb were tested, and the correlation coefficients of the 67 individual samples were calculated. To assign mRNA gene positions, we selected mRNA TSS positions according to the following criteria, in contrast to Kristjánsdóttir et al.^21^ which considered all annotated TSSs for each mRNA. First, we selected annotated mRNA TSSs that overlapped with a promoter tTRE within 250 base pairs of distance. Then, we further selected the mRNA TSS with the highest PRO-cap expression level within the same mRNA transcripts. Therefore, 1 representative mRNA TSS position was selected for each gene. Correlation coefficients were calculated as described above for tTRE-tTRE co-expression.

### Analysis of the ENCODE factor dependencies in tTRE co-expression

The top 5 percentile, as well as the median, of the correlation coefficients in the variable interval bins were used as the indicators of tTRE co-expression. This correlation coefficient percentile serves as a step function of the distance, and we refer to it as the correlation decay plot. The correlation decay plots were generated by subsetting the tTRE pairs by their distance with 1000 tTRE pairs per bin as described in the ‘Distance-dependent pair-wise co-expression analysis of tTREs’ section. The top 5 percentile of the correlation coefficient was generated from the pairwise correlation coefficients between any pair of tTREs within 200 kb distance, resulting in a total of 192.4 thousand total pairs. The Area Under the Curve was calculated for the 5th percentile step function we generated between 0 and 200 kb range. The same ∆AUC was generated using the median step function to validate the ∆AUC based on the 5th percentile step function to evaluate the consistency of the metric and liability to noises.

∆AUC values were calculated to evaluate whether TFs located either between the tTREs or at the tTREs affected the correlation decay plots. We used the ENCODE FACTORBOOK binding sites in the representative LCL GM12878- to separate the 192.4 thousand tTRE pairs between 0 and 200 kb distances into 2 groups based on the respective TF binding or intersection statues, and calculated the difference between the area under the correlation decay curves in the 0–200 kb distance range (∆AUC). Both the 5th percentile and the median correlation decay step functions were used, and ∆AUCs from the 5th percentile and the median values correlated well across TFs (Supplementary Fig. 4a,b). For the TF intersection analysis, we compared 0 or 1 TF intersection against 2 or more TF intersections between the tTRE pairs, as there were not enough tTRE pairs with 0 TF intersection in longer distance ranges for statistical comparisons.

The statistical significance of ∆AUC values on TF intersection and occupancy was estimated using a bootstrapping randomization strategy. We also considered whether the number of factor binding sites affects the dispersion of ∆AUC values, by using a different number of mock sites to simulate the effects of different numbers of TF binding sites on the ∆AUC calculation, especially for the TFs with fewer or higher binding sites. The expected mean and standard deviation of ∆AUC with the randomized sets allowed us to estimate the p-values and FDR of ∆AUC in the TF intersection and occupancy plots.

Specifically, the significance of the ∆AUC was estimated by comparison with the background distribution of ∆AUC which was generated by randomly shuffling the genomic locations of the factor binding sites. We calculated the background ∆AUC distributions by 1000 permutations of 5000 to 70,000 randomly shuffled ENCODE factor binding sites, maintaining the same tTRE expression vector levels across individuals. The background ∆AUC distributions followed a normal distribution and were dependent on the number of binding sites. We approximated the expected mean and standard deviation (sd) of the ∆AUC as a function of the actual number of binding sites for the specific factor, and used this to generate a z-score and p-value of the ∆AUC between correlation decay curves enriched or depleted with the factor binding sites (Supplementary Fig. 4c,d). For example, CTCF contains 41,465 ChIP-seq binding sites^28^, and the ∆AUC = + 27.8 between more than 2 CTCF sites or less than 1 site intersecting a pair of tTREs. The background distribution of ∆AUC with 40,000 sites intersecting tTREs is + 6.41 ± 2.56 (mean ± sd), and we obtained the z-score = + 8.34 and p = 7.26 × 10^−17^ for CTCF intersections affecting correlation decay plots. The intersection and the occupancy scores of all other 76 factors available for GM12878 in FACTORBOOK were calculated in this way. Only factors with a sufficient number of binding sites in each category were reported. These p-values and z-scores were used in the clustering analysis to cluster the TFs.

### Hi-C analysis

As a representative LCL, GM12878 Hi-C data were obtained from the 4D Nucleome Consortium public database (accession # 4DNFIXP4QG5B). To extract the Hi-C contact frequency data, we first converted our tTRE coordinates from the hg19 to the hg38 reference genome used by the 4D Nucleome Consortium using NCBI liftover. Contact frequencies from the regions 500 kb upstream and downstream of the tTRE positions were extracted from the Hi-C mcool data using the cooler package (https://cooler.readthedocs.io/en/latest/index.html) at 5 kb resolution. The extracted contact frequency table was used to query the exact contact frequency between tTRE pairs within 500 kb distance. The distance-dependent decay, ENCODE TF dependency, and the ∆AUC analyses were performed in the same way as the co-expression correlation analyses, by using the contact frequency values and the log of contact frequencies instead of the co-expression coefficients, and using the same tTRE pair classifications. Background subtraction was performed on the linear values of Hi-C contact frequencies prior to log transformation.

### Strand-specific co-expression analysis for tTRE nascent transcription at adjacent sites

For each tTRE, the nearest adjacent downstream tTRE was identified and a linear regression between its strands was computed across the samples according to the following categories: Sense (plus–plus, minus–minus), Antisense convergent (plus–minus), and Antisense divergent (minus–plus). Pairs of tTREs were binned based on the distance between them, and the distributions of Pearson correlation coefficients were compared between the categories. Pairs within 250 bp were not included to avoid the possibility of counting the same reading in both tTREs. A total of 21,486 pairs of adjacent tTREs (16,857 for enhancers only) within 10 kb distances were compared. To compare distance dependence, LOESS fits were generated by taking 200 local data points on the distance axis for each data point to calculate the local polynomial regression curves (Supplementary Fig. 6a).

For the adjacent vs interleaved tTRE analysis, two sets of tTRE pairs were selected, those that were immediately neighboring (adjacent) or those that contained one other tTRE in between (interleaved). The LOESS fits of their PRO-cap co-expression correlation coefficients or Hi-C contact frequencies in 1 kb resolution were generated.

### Supplementary Information

Supplementary Information.

Supplementary Table S1.

Supplementary Table S2.

Supplementary Table S3.

## Data Availability

The data can be accessed through the GEO (GSE110638). The datasets generated and analyzed in the current study are available in the Github repository, [https://github.com/sl2665/procap-network]. ChIP-seq data was obtained from ENCODE defined Transcription Factor ChIP-seq Peaks (338 factors in 130 cell types) ENCODE3 Nov. 2018. We have selected defined ChIP-seq peaks from GM12878 cell line dataset. Hi-C data was obtained from 4D Nucleome Consortium database (dataset id: 4DNFIXP4QG5B).
