# Supplementary material for: eRNA co-expression network uncovers TF dependency and convergent cooperativity
Source: Sci Rep. 2023 Nov 4;13:19085. doi: 10.1038/s41598-023-46415-2 (PMC10625640; doi:10.1038/s41598-023-46415-2)
Supplement: Supplementary file 1 — Supplementary Information. [file 41598_2023_46415_MOESM1_ESM.pdf]

Supplementary Figure 1

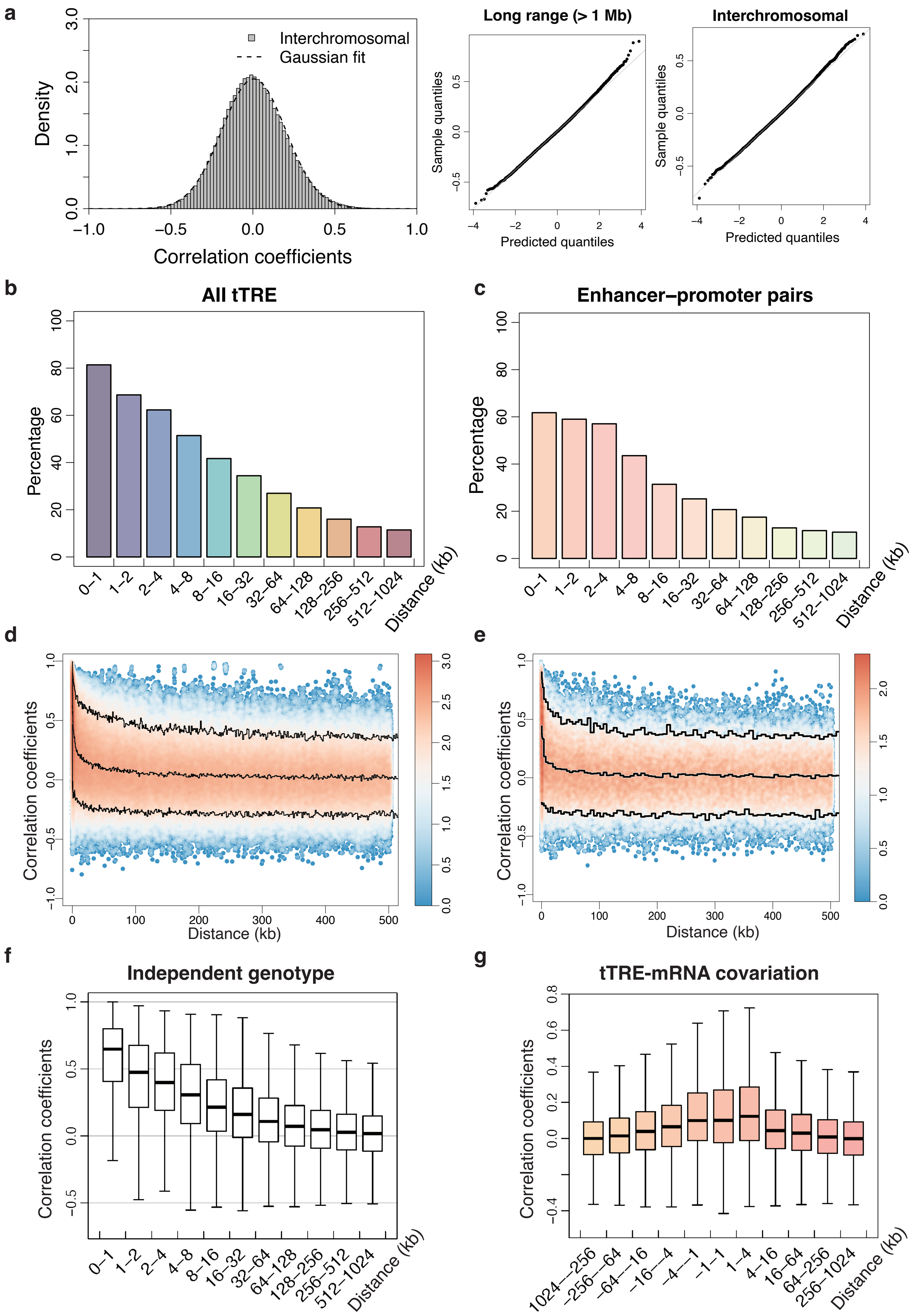

Supplementary Figure 1. Co-expression of tTREs dependent on the distance.

(**a**) Distribution of correlation coefficients between interchromosomal tTRE pairs. The trace line indicates a Gaussian fit of the distribution (left); Q-Q plots of distant intrachromosomal correlation coefficients (>1Mb) and interchromosomal correlations (right). (**b**) Fraction of significantly correlated tTRE pairs (FDR < 0.05) for all tTRE pairs. (c) Fraction of significantly correlated tTRE pairs (FDR < 0.05) for enhancer-promoter pairs. (**d**) Distribution of correlation coefficients among all tTREs depending on their relative positions. Density scatter plots represent the distribution of the correlation of all tTRE pairs, and the 3 lines represent the quantile traces of 5 percentile, median and 95 percentile of the correlation coefficients. (**e**) Distribution of correlation coefficients among Enhancer-promoter pairs depending on their relative positions. Density scatter plots represent the distribution of the correlation of all tTRE pairs, and the 3 lines represent the quantile traces of 5 percentile, median and 95 percentile of the correlation coefficients. (**f**) Box plots of correlation coefficients of tTRE co-expression in tTREs with independent genotypes (genotype correlation <0.05) to exclude tTRE pairs in linkage disequilibrium. (**g**) Scatter plots of correlation coefficients between mRNA levels and tTRE PRO-cap levels plotted against the distances between tTRE and mRNA TSS.

Supplementary Figure 2

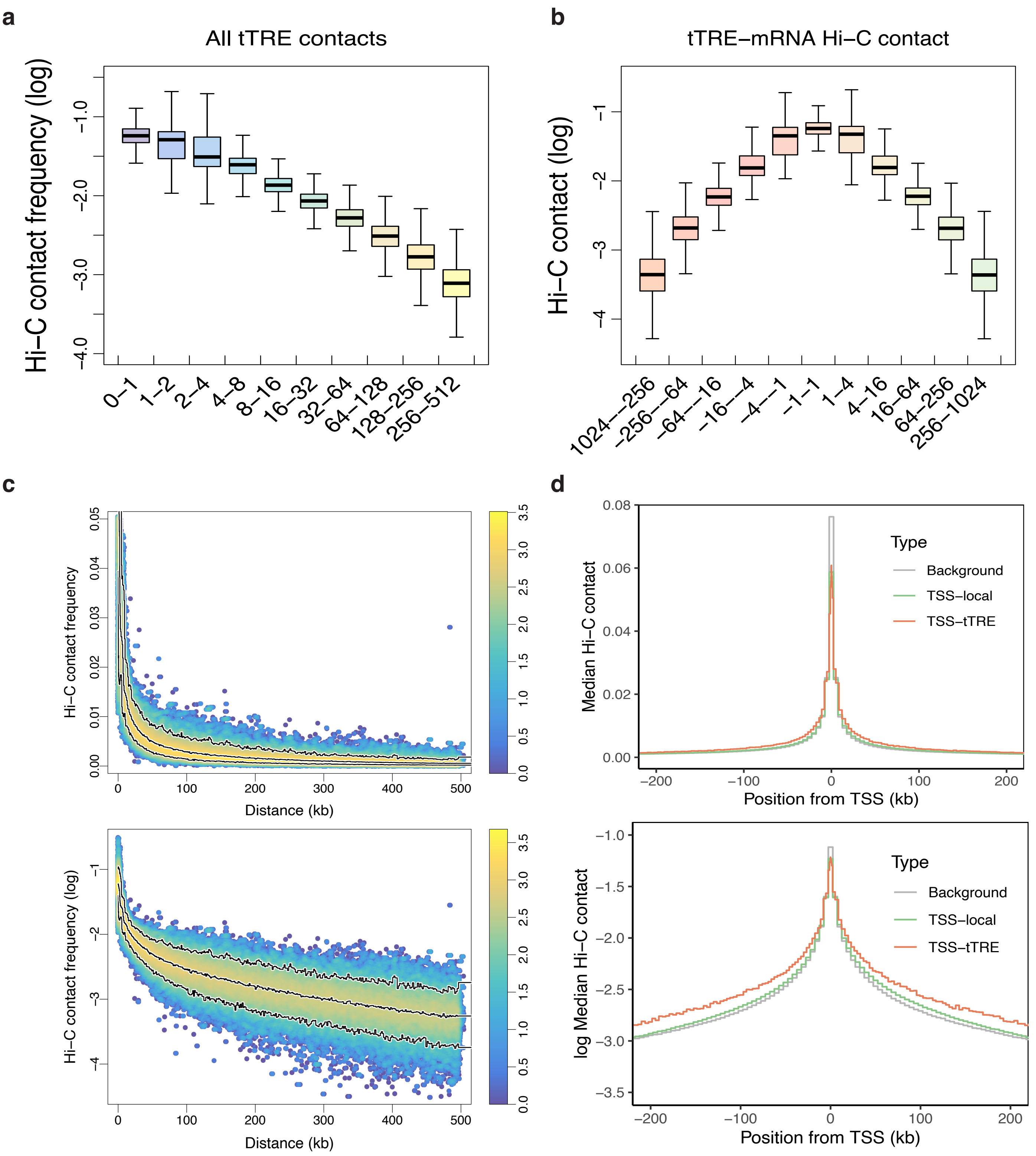

Supplementary Figure 2. Co-expression of tTREs dependent on the distance in Hi-C contact plane. (**a**) Boxplot of Hi-C contacts between all tTRE pairs. (**b**) Boxplot of Hi-C contacts between tTRE -mRNA pairs. (**c**) Traced scatterplot of Hi-C contacts between all tTRE pairs; alternative representation of Hi-C contacts with log value (lower panel). (**d**) Quantile tracing of Hi-C contacts compared to mRNA-PRO-cap correlation of co-expressions. Traces of Hi-C contacts between random positions (background), gene TSS to all local regions (TSS-local), or gene TSS to tTREs (TSS-tTRE); alternative representation of Hi-C contacts with log value (lower panel).

Supplementary Figure 3

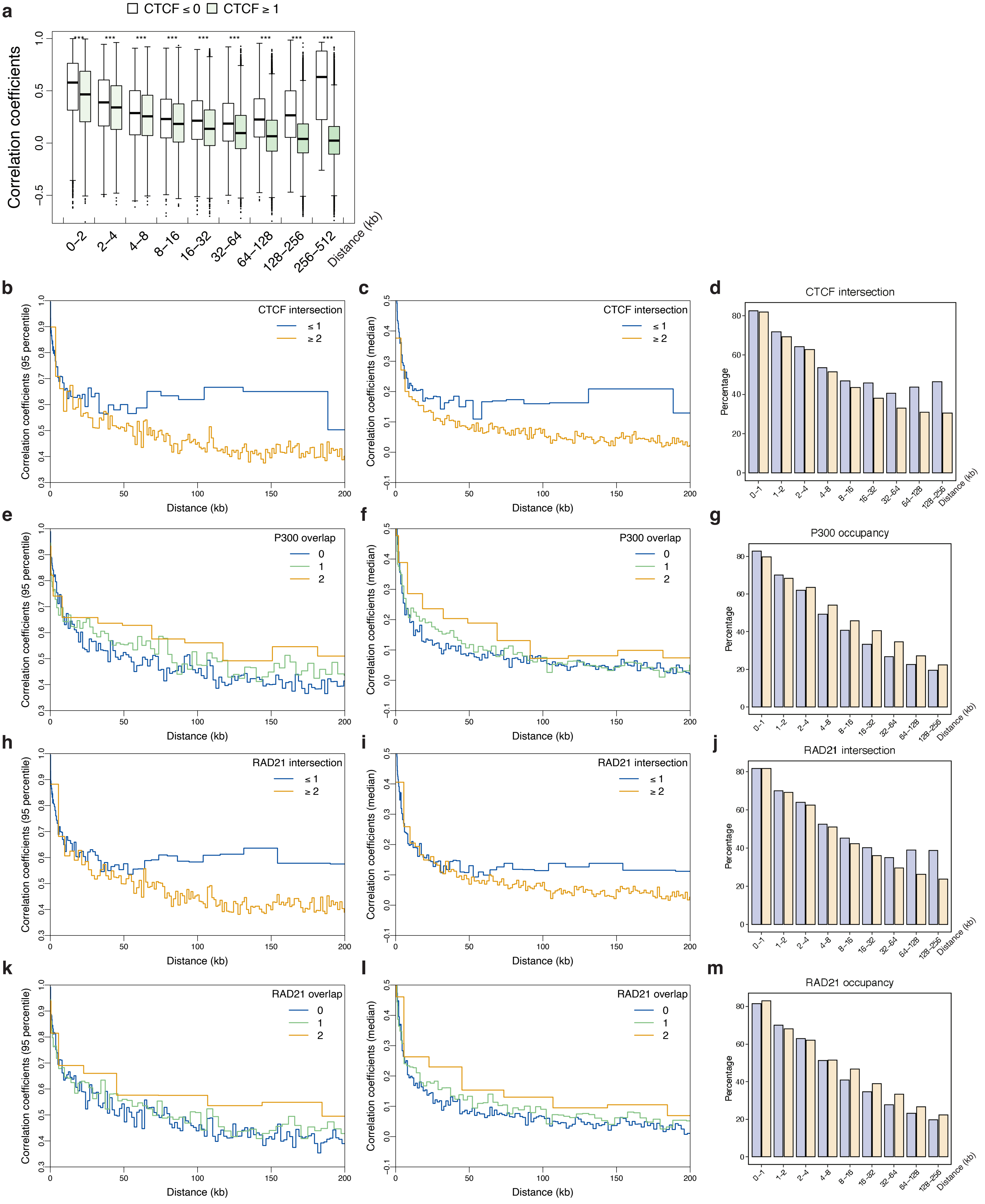

Supplementary Figure 3. Association between Transcription Factor binding sites and variations in the co-expression of tTRE using PRO-cap. (**a**) Boxplot of correlation coefficients between tTRE pairs grouped by 1 or more CTCF intersection and no-intersection, binned by the distance. The numbers of tTRE pairs in each distance bin are indicated in Supplementary Table 3. (**b-d**) Association between intersection by an insulator and decrease in AUC. (**b**) Distributions of co-expression of tTRE using PRO-cap for tTRE pairs intersected by 2 or more CTCF sites and 1 or less, binned by distance in 95 percentile level. (**c**) Distributions of co-expression of tTRE using PRO-cap 2 or more CTCF sites and 1 or less, binned by distance in median level. (**d**) Fraction of tTRE pairs with statistically significant interactions (FDR < 0.05), observing the distance-dependent decay patterns for intersection analysis for CTCF. (**e**) Distributions of co-expression for tTRE pairs using PRO-cap. 95 percentile traces occupied by 0,1, or 2 P300 sites binned by distance. (**f**) Distributions of co-expression for tTRE using PRO-cap, median traces occupied by 0,1, or 2 P300 sites binned by distance. (**g**) Fraction of tTRE pairs with statistically significant interactions (FDR < 0.05), observing the distance-dependent decay patterns for occupancy analysis for P300. (**h-j**) Plots of RAD21 intersection, generated in the same manner as the panels a-c. (**k-m**) Plots pf RAD21 occupancy, generated in the same manner as the panels e-g. Plots pf RAD21 occupancy, generated in the same manner as the panels e-g. Color scheme: blue - 0-1 TF intersected TF unbound; orange - 2 or more TF intersected / TF bound (panels **b-m**).

Supplementary Figure 4

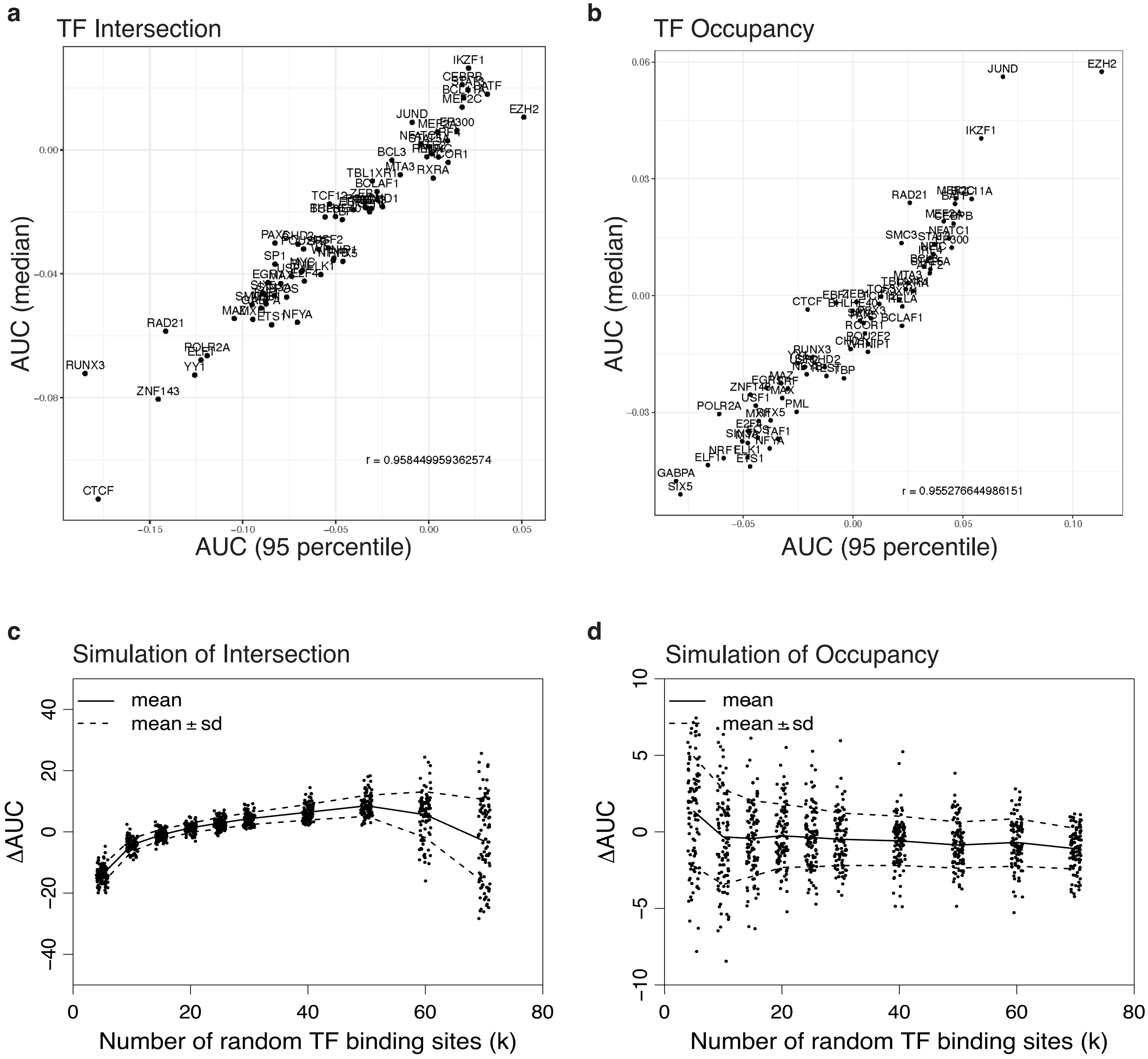

Supplementary Figure 4. Validation of TF dependency AUC metrics by comparing different percentiles and randomization strategies to calculate statistical significance. (**a**) Comparison of the AUC generated from the median step function of all random Transcription Factors to the AUC generated from the 95th percentile step function showing simulation of intersection. (**b**) Comparison of the AUC generated from the median step function of all random Transcription Factors to the AUC generated from the 95th percentile step function showing simulation of occupancy. (**c**) Simulated distribution of randomized AUC values in intersecting TF binding sites depending on the number of TF binding sites. (**d**) Simulated distribution of randomized AUC values in occupying TF binding sites depending on the number of TF binding sites.

Supplementary Figure 5

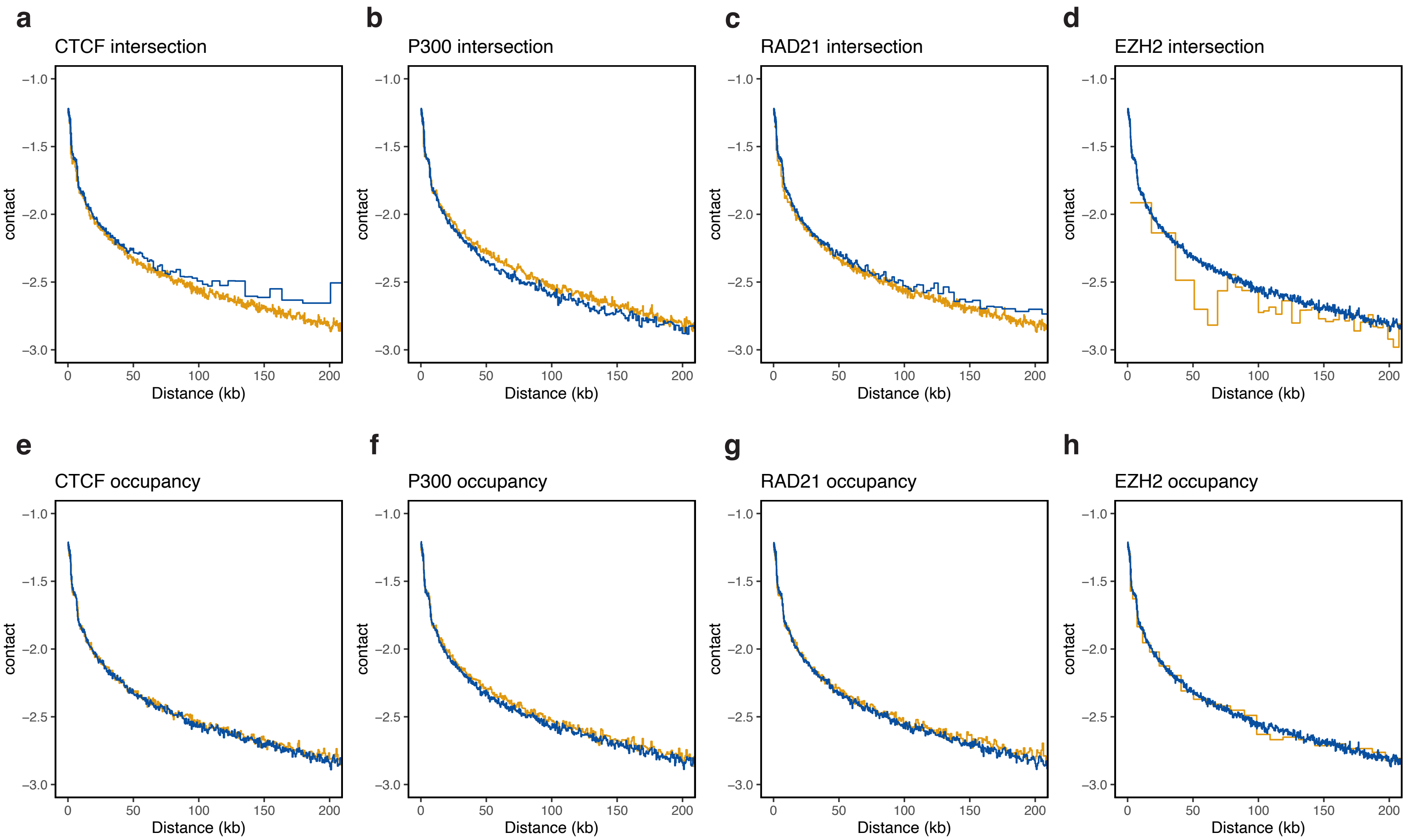

Supplementary Figure 5. Distance-dependent Hi-C contact frequency traces grouped by TF intersection or occupancy. (**a**) Distance-Hi-C contact frequency plot traces (median of 1,000 tTRE pairs in each step bin) grouped by CTCF intersection between the tTREs. Orange lines: 1 or 2 CTCF intersections, blue lines: 0 or 1 CTCF intersections. (**b-d**) Same distance-dependent Hi-C contact frequency traces for P300, RAD21, and EZH2, respectively. (**e**) Distance-Hi-C contact frequency plot traces (median of 1,000 tTRE pairs in each step bin) grouped by CTCF occupancy at the tTREs. Orange lines: 1 or 2 CTCF occupancy, blue lines: no CTCF occupancy. (**f-h**) Same as panel e, for P300, RAD21, and EZH2, respectively.

Supplementary Figure 6

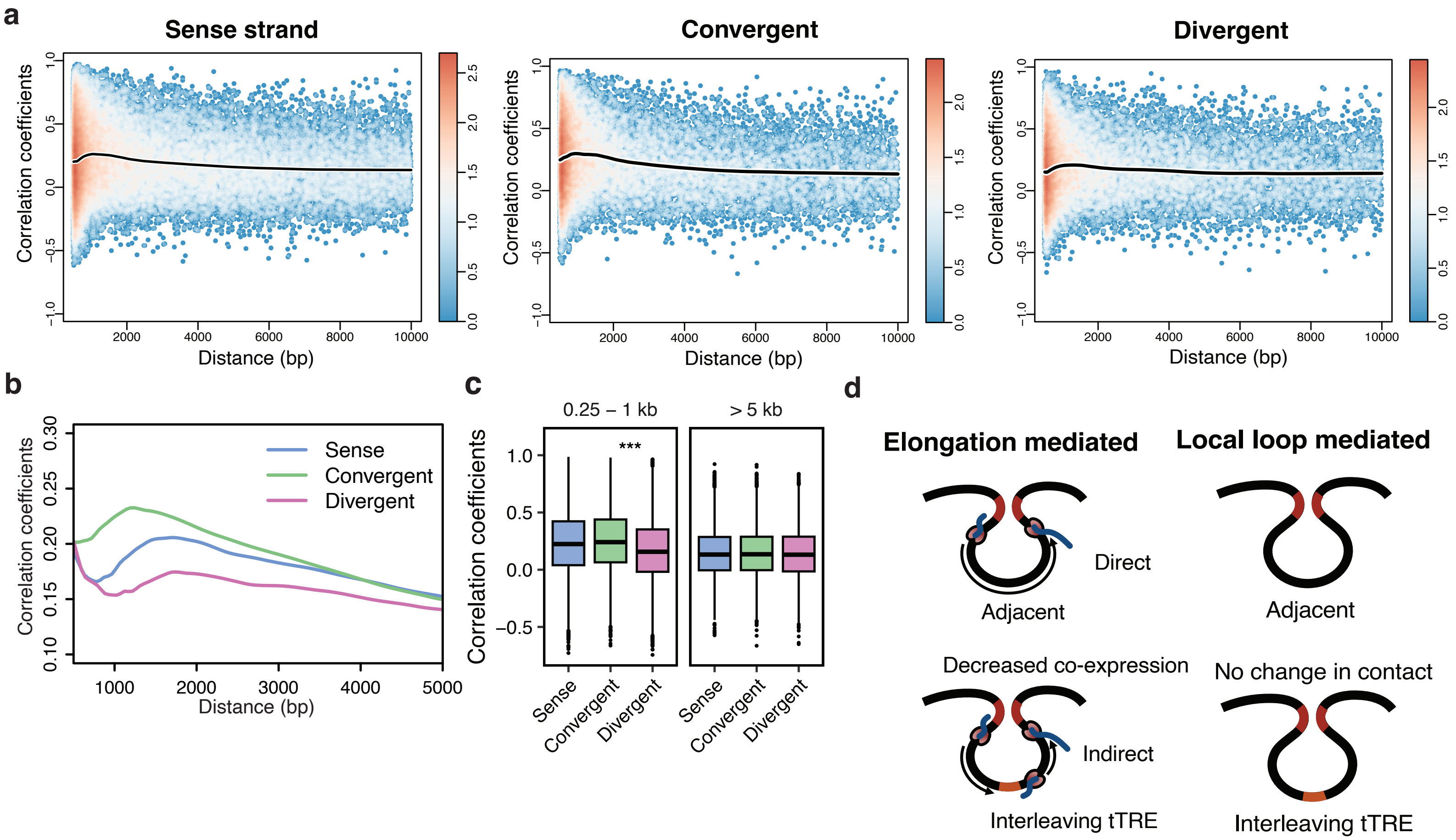

Supplementary Figure 6. Directional co-expression of neighboring tTREs. (**a**) Scatterplot of strand specific co-expression of the neighboring convergent and divergent strand nTSSs of the tTREs along the distance axis. Trace line represents a locally weighted scatterplot smoothing (LOESS) fit of the mean distribution. (**b**) Loess fit for the correlation coefficient of adjacent enhancers (only) in sense, convergent or divergent orientations. (**c**) Strand comparisons, for all adjacent tTREs that are either between 250 bp and 1 kb apart or more than 5 kb apart. The distribution of correlation coefficients for sense, antisense convergent (conv), and antisense divergent (div) strands is plotted as a boxplot (*** P < 2.2 x 10-16, Wilcoxon Rank Sum Test). (**d**) Models explaining eRNA correlation by interleaved tTREs. Left: The diagram illustrates the modeling of eRNA correlation mediated by Pol II elongation. Neighboring eRNAs are more correlated when Pol II elongation can directly reach the neighboring enhancer (top). The presence of an interleaved enhancer disrupts the direct reach of Pol II elongation and leads to indirect association, resulting in decreased correlation (bottom). Right: The diagram illustrates the modeling of eRNA correlation mediated by local loops. Neighboring enhancers interact through local loops (top). The presence of interlacing enhancers is not associated with changes in contract frequency in Hi-C data (bottom).This model supports the hypothesis that convergent eRNA correlation is mediated by a non-looping mechanism, which we propose is dependent on Pol II elongation (see Discussion).

Supplementary Table Legends

Supplementary Table 1. Number of PRO-cap reads mapped to the genome, promoters, and enhancers. The number of unique molecular identifier (UMI) tagged PRO-cap reads in 76 partially replicated PRO-cap data in Lymphoblastoid Cell Lines (LCLs) from 67 individuals from the Yoruban population (YRI). Cell lines are indicated by GM-number, and replicated data are tagged with "r" at the end of sample names. mappedReads: UMI mapped reads to the whole genome; enhancerReads: UMI reads mapped to tTREs in enhancer regions; promoterReads: UMI reads mapped to tTREs in promoter regions that overlap with annotated refSeq TSSs; enhancerPercent: percentage of enhancerReads/mappedReads; promoterPercent: percentage of promoterReads/mappedReads.

Supplementary Table 2. The number of Chromatin Immunoprecipitation (ChIP-seq) peaks used in TF binding site analyses. These peaks were pre-defined from the ENCODE Factorbook repository in the representative LCL (GM12878)[27], and were used to determine the effect of TF binding on distance-dependent tTRE correlation trends (relevant to Figures 3 and 4).

Supplementary Table 3. The number of tTRE pairs in each distance bins. Number of tTRE pair events in each distance bins to evaluate the significance of statistical tests (relevant to Figures 3a-c, 4a-c, and Supplementary Figure 3a).
